# Toward “Age-Friendly Slums”? Health Challenges of Older Slum Dwellers in Nairobi and the Applicability of the Age-Friendly City Approach

**DOI:** 10.3390/ijerph14101259

**Published:** 2017-10-20

**Authors:** Isabella Aboderin, Megumi Kano, Hilda Akinyi Owii

**Affiliations:** 1 African Population and Health Research Centre, Nairobi 10787-00100, Kenya; iaboderin@aphrc.org (I.A.); howii@aphrc.org (H.A.O.); 2 Centre for Research on Ageing, University of Southampton, Southampton SO17 1BJ, UK; 3 OPTENTIA Research Focus, North West University, Vanderbijlpark 1900, South Africa; 4 Centre for Health Development, World Health Organization, Kobe, Hyogo 651-0073, Japan

**Keywords:** urban, slums, age-friendly, ageing, sub-Saharan Africa

## Abstract

A majority of urban residents in sub-Saharan Africa (SSA) and other developing regions live in informal settlements, or slums. Much of the discourse on slum health centres on younger generations, while an intensifying agenda on healthy ageing as yet lacks a systematic focus on slums. Similarly, the global age-friendly cities (AFC) movement does not, thus far, extend to slums. This paper examines the particular challenges that a slum-focused age-friendly initiative in SSA may need to address, and the relevance of present AFC indicators and domains for initiatives to advance the health and well-being of older slum dwellers. The analysis builds on the case of two slum communities in Nairobi, Kenya. It analyzes two bodies of relevant evidence from these settlements, namely on the health and social circumstances of older residents, and on the local application and measurement of AFC indicators. The findings point to a set of unsurprising, but also less obvious, core health and social adversities that an age-friendly initiative in such settlements would need to consider. The findings show, further, that the current AFC domains and indicators framework only partly capture these adversities, but that there is potential for adapting the framework to be meaningful for slum settings. The paper concludes by underscoring the need for, and opportunities inherent in, the pursuit of an “age-friendly slums” initiative going forward.

## 1. Introduction

Sub-Saharan Africa (SSA) is rapidly urbanizing, with the share of the region’s population residing in urban areas expected to rise from 37.9% today to 55% by 2050 [1]. An estimated 61% of SSA’s urban inhabitants are clustered in informal settlements, a higher proportion than in any other world region, and the number of such slum residents is set to increase dramatically in coming decades [2,3].

Public health and policy debates increasingly recognize that slum environments, characterized by a lack of access to safe drinking water, inadequate sanitation, pollution, poor housing and overcrowding, as well as social deprivation and threats, pose profound risks to well-being. At least in part, these risks are driven by neighbourhood effects of the spatially-distinct slum communities, thus going beyond those associated with urban living and poverty alone [4,5,6].

The projected growth in slum populations intersects critically with population aging: slums are becoming an ever more salient context within which Africans grow older and spend all or part of their later lives. Thus far, however, discourses on the health of people who live in such settlements have focused largely on children, adolescents, and reproductive-age adults [4,7]. Health issues of older slum dwellers have received little consideration. Similarly, an intensifying World Health Organization (WHO)-led agenda on “healthy ageing” has not, thus far, paid systematic attention to slum environments [8]. These gaps exist despite the fact that slum exposures may have particularly far-reaching health consequences in later life. Moreover, the health of older slum residents may have direct influences on the prospects of younger generations in such settings as they often cohabit with, or care for children and youth [9,10].

A focused effort may be needed to better understand and to forge solutions to the most critical health challenges facing older slum dwellers. Indeed, such an endeavour would capture the nexus of two vital global development and urban agendas. These are: (i) the Sustainable Development Goals (SDG), which enshrine the imperatives of ensuring health and well-being at all ages, including through upgrading services and conditions in slums [11]; and (ii) the New Urban Agenda, with its explicit focus on improving well-being and opportunities for slum populations [12]. Corresponding regional agendas are the African Union’s overarching Agenda 2063 [13] and the African Urban Agenda [2] (see Box 1).

Box 1 **Key global and regional development and urban agendas relevant to older slum dwellers’ health.**

***1. Sustainable* *Development Goals***

The framework of sustainable development goals (SDG), adopted by the United Nations General Assembly in 2015 as an overarching agenda for global development to 2030, centres on a quest to ensure that all human beings “can fulfil their potential in dignity and equality and in a healthy environment”. Particularly pertinent goals to the health of urban slum residents include:

Goal 3: Ensure healthy lives and promote wellbeing for all at all ages.
  *Target* *3.4*: By 2030, reduce by one-third premature mortality from noncommunicable diseases through prevention and treatment and promote mental health and well-being.
  *Target* *3.8*: Achieve universal health coverage, including financial risk protection, access to quality essential health-care services and access to safe, effective, quality and affordable essential medicines and vaccines for all.
Goal 11: Make cities and human settlements inclusive, safe, resilient and sustainable.
  *Target* *11.1*: By 2030, ensure access for all to adequate, safe and affordable housing and basic services and upgrade slums.

***2. New* *Urban Agenda***

The global New Urban Agenda (NUA) seeks to readdress the way cities and human settlements are planned, designed, financed, developed, governed, and managed, in order to end poverty and hunger, reduce inequalities, promote inclusive and sustainable economic growth, achieve gender equality, foster the empowerment of all women and girls, improve human health and well-being, promote resilience and protect the environment. As part of this endeavour the NUA emphasises a need for an upgrading of slums and an enhancement of the basic services provided within them.

***3. Agenda* *2063***

The African Union’s Agenda 2063 offers an overarching blueprint for Africa’s development over the coming five decades. Informed by lessons learnt from past African Union plans and commitments, Agenda 2063 envisions, among others, an Africa whose people have high standards of living, quality of life and sound health and well-being, and whose human rights are respected. In its call to action, the Agenda makes explicit reference to those living in slums, positing a need for action to improve the livelihoods of this large population.

***4. African* *Urban Agenda***

Complementary to the New Urban Agenda, the initiative toward an African Urban Agenda seeks to offer a framework for the pursuit of sustainable urban development that is responsive to the specific multiple interlocking dynamics of distorted urbanisation across the continent. In this regard the framework (i) proposes investments in enhancing basic services and education and health as the top two priority areas for action; and (ii) underscores the imperative of such investments in slum populations.

The Age-Friendly Cities (AFC) initiative of the WHO constitutes an increasingly prominent global movement on advancing the well-being of older adults living in urban areas, broadly [14,15]. The initiative aims to foster inclusion, participation, security, and support for all older urban residents in order to enhance their well-being and positive contributions into oldest ages [15]. The AFC approach is being implemented in a rising number of localities worldwide, reflecting a growing recognition of the juncture between global urbanization and aging trends. As the movement expands, WHO has developed a suggested framework of core AFC domains and indicators to support the assessment of the age-friendliness of communities, and to monitor and evaluate progress in improving it [16]. The indicator domains include neighbourhood walkability, accessibility of public spaces and buildings, as well as of public transportation vehicles and stops, affordability of housing, positive social attitudes toward older people, engagement in volunteer activity, paid employment, engagement in socio-cultural activities, availability of social and health services, economic security, participation in local decision-making, availability of information, quality of life, and equity. The framework, which draws on a literature review, international expert consultations, and earlier work, is intended as a resource and tool for communities wishing to initiate, or consolidate existing, AFC projects [16]. Thus far, however, the global AFC movement includes neither a locality in Africa nor an explicit focus on slum environments. 

This situation raises key questions about (i) the particular health-related issues or challenges that a slum-focused age friendly cities initiative, particularly in SSA, would need to consider; and (ii) the extent to which the AFC approach may offer an appropriate basis, and its suggested core indicators and domains a useful frame, for initiatives to advance the health and well-being of older adults living in such settlements.

Our aim in the remainder of this article is to reflect on, and propose initial answers to these queries. To do so we draw, opportunistically, on the case of two specific slum communities, Korogocho and Viwandani, in Nairobi, Kenya, which form one of only two urban health and demographic surveillance systems (HDSS) in SSA [17,18] and for which two relevant bodies of evidence are available. One is a spectrum of information on the life and health circumstances and contexts of older slum residents, generated by routine HDSS data rounds and a range of qualitative and quantitative studies that have been nested within the HDSS in recent years. Key examples include a survey on urbanization, population, and health dynamics (UPHD), as well as an investigation of later life resilience [10,19,20,21,22,23,24].

A second, pertinent body of evidence entails findings on the local application of the AFC core domains and indicators in the two slum communities. Such findings were produced by a 2015 pilot assessment of the WHO AFC Indicator Guide undertaken in Korogocho and Viwandani, among 15 localities worldwide [16,25]. Broadly, the Guide aims to support the planning and implementation of AFC initiatives and associated research by setting out the core dimensions and indicators, as well as direction on their selection and use in the field [16]. The 15 pilot sites globally spanned a wide spectrum of geographical, socio-cultural, linguistic, and ageing-related contexts and included: La Plata, Argentina; Banyule, Australia; Hong Kong, China; Shanghai, China; Dijon, France; New Delhi, India; Tehran, Iran; Udine, Italy; Nairobi, Kenya; Tuymazy, Russia; Bilbao, Spain; Bowdoinham, USA; Washington, DC, USA; New Haven, USA; and Fishguard-and-Goodwick, UK. An evaluation of the global pilot study has been published elsewhere [26].

## 2. Materials and Methods

We undertook a review and further analysis of existing evidence as follows. Available findings from prior quantitative and qualitative studies on the health, situation, and broader contexts of older adults in Korogocho and Viwandani was examined in order to distil a profile of the general circumstances of such older slum residents and the likely key health challenges facing them.

This was followed by a review of the results of the 2015 AFC Indicator Guide pilot study, which had sought to determine the “age-friendliness” of the two communities as assessed against a set of core AFC indicators. Guided by a standard cross-site protocol, the pilot had developed locally-tailored operational definitions of the indicators. To populate the indicators, the study had then employed secondary analysis of two existing quantitative data sources, as well as the small-scale collection of primary qualitative data. The existing data sources that were used in the 2015 pilot comprised (i) information on household livelihoods and well-being extracted from the 2013 Nairobi Urban Health and Demographic Surveillance System (NUHDSS); and (ii) data on the situation and wellbeing of older residents extracted from a 2009 survey on Urbanization, Poverty and Health Dynamics (UPHD), which used questionnaire modules from the WHO Survey on Adult Health and Ageing. Qualitative data collection in the 2015 pilot study entailed (i) on-the-ground observation within communities to map environmental conditions, such as walkability, accessibility, or the existence of particular services in some instances complemented by consultation with relevant role players; and (ii) two focus groups held with community-dwelling older men and women. The specific approaches used for the secondary analysis and qualitative exploration have been described elsewhere [25].

Data analysis in the 2015 pilot generated overall measures of, and perspectives on, indicator outcomes, as well as potential gender, age, and socio-economic inequities in them. Equity analyses calculated population attributable risk (PAR) and PAR% for older women compared to older men; younger old (60–69 years) compared to older old (70 years and above) adults, and those residing in relatively poorer, compared to those in less poor, households (the relative poverty of a household was assessed using an asset-based index, which captures a household’s possession of the following: vehicle, motorcycle, bicycle, refrigerator, television, radio/stereo, DVD/VCD/VCR, sewing machine, electric iron, fan, telephone/mobile phone, electric/gas stove, sofa set, table, torch, kerosene lamp with glass, kerosene stove, wall clock, mattress, blankets, and bed) [25].

## 3. Results

### 3.1. Review of Available Evidence: Circumstances and Key Health Challenges of Older Slum Residents

Slum settlements like Korogocho and Viwandani are replete with people of low educational attainment who are unemployed, poorly-paid casual labourers, or informal and low-capacity retailers. Despite recent slum upgrading interventions focusing on issues of tenure and infrastructure especially in Korogocho [7], the communities remain exposed to overcrowding, both within and between households, and experience raised levels of social ills, such as alcohol and substance abuse, domestic violence, and crime [7,27]. For many rural to urban migrants, such settlements are a first stop in their transition to city life. For many, they are also a last stop as migrants age and grow old within the slums, often despite an envisaged return to their rural homes [21].

Routine NUHDSS data suggests that the number of older slum dwellers living in the two communities, who typically moved to the city in the 1970s and 1980s, is considerable. June 2017 estimates show Korogocho, with a total population of 30,376—to be home to close to 1102 older adults; with respective figures for Viwandani of 52,852 and 578 [28]. In both settlements, moreover, the number of older residents has risen sharply over the past decade—by 53% in Korogocho and 138% in Viwandani between 2003 and 2014—at rates that have surpassed the communities’ overall population growth rates of 6.1% and 23.7%, respectively [25].

A number of markers point to key features of older slum dwellers’ social and economic circumstances. One is pervasive economic strain: 43% of households containing an older person live in absolute poverty (on less than 1.90 USD/day) [28]. As in several other SSA countries [29], a social protection scheme targeting vulnerable older adults—the Government of Kenya’s Older Persons Cash Transfer Program (OPCTP)—now exists. However, only a relatively small number of older adults in the two communities (estimated at 320 for financial year 2015/16) benefit from it [30]. Another aspect is the extensive co-residence of older adults with younger generations: in Korogocho, close to 40% of older women and 30% of older men live with at least one child below the age of 15, and similar shares reside with at least one youth aged between 15 and 24 years. The corresponding estimates for Viwandani are slightly lower [25]. More detailed information on older slum residents’ household composition and flows of support within or between them is not available at present. However, as has been described for other SSA contexts [31,32], qualitative insights from the two communities suggest that older adults’ co- or proximate residence with younger generation kin often entails caregiving responsibilities, especially for grandchildren whose parents are deceased, absent, or incapable due to sickness, alcohol abuse or other factors.

Within this context, available qualitative and quantitative evidence suggests four likely central challenges to older people’s health in the two communities. The first, highlighted by UPHD data, is a prominence of musculoskeletal disease (MSK) as the most commonly reported serious health problems for older adults in the slum communities [33]. This broadly echoes findings on the prevalence of later-life MSK in other SSA settings [9,34,35]. The severity of the impacts of MSK for slum dwellers is indicated in its observed links to impaired functioning and poorer subjective well-being [33] and in the qualitative accounts of later-life resilience study respondents. In these accounts older slum residents highlight the physical suffering from untreated musculoskeletal conditions, as well as the limited mobility and the curtailed opportunities for work, social engagement, and resilience that it implies, as a central, daily adversity in their lives [24,36].

A second key challenge to older adults’ health in the slum communities, again highlighted in the study on later-life resilience, is, for many, a sustained, often acute stress and anxiety about the poor prospects of their children and grandchildren, or the threats of violence, disrespect, or aggression from such younger generation kin. Such stress appears exacerbated by the crowding and lack of space within households and by older slum dwellers’ aforementioned care responsibilities for younger generation kin. Beyond its mental or emotional costs, such “generative anxiety” is experienced by older slum dwellers as a direct threat to their physical health and survival [24,36].

A third major health adversity, which exacerbates the prior two, is older slum residents’ circumscribed access to requisite health care. Emphasized by respondents in the later-life resilience study, this gap is borne out by UPHD study data: almost half (43%) of older adults report not obtaining health care when they required it in the past 12 months [24,36,37]. Likely drivers of such circumscribed health care access include (i) a dearth of public health facilities in settlements designated as “informal” [38]; and (ii) cost barriers to the use of private services and medicines [9,24].

A final, core challenge relates to the poor built environment within which older slum residents live. Older respondents’ accounts in the resilience study bemoan their substandard housing with inadequate access to basic amenities. The scale of privation in this regard is underscored by routine NUHDSS data, which show only 6% of households to have access to piped water and more than half (51%) to share, often faulty, toilet facilities [28]. Defective electric connections that lead to electrocutions and fires, as well as poor neighbourhood garbage management, contribute hazards to health [7].

### 3.2. AFC Indicator Guide Pilot: Pervasive Age “Unfriendliness”

Table 1 presents results of the operationalization and measurement of the AFC core indicators in Korogocho and Viwandani. It shows that indicators on the built environment indicators were measured directly using on-the-ground observation and mapping, while most other indicators were estimated through proxy measures, mainly using data from the NUHDSS or UPHD.

Measures of the indicators showed a starkly unfavourable physical environment characterised by poor neighbourhood walkability, and severely limited accessibility of public spaces, buildings, and transportation vehicles and stops. Of 17 roads in Viwandani none are tarmacked; of 23 in Korogocho, the few that are tarmacked have either partial or no walkways. None are considered to be easily accessible for pedestrians, especially for older people with mobility challenges. Public buildings in Viwandani are three local administration offices, three main churches, and two public primary schools. In Korogocho these comprise two local administration offices, a public hall, seven village elders’ offices, one public primary school, and one church. None have accommodations for wheelchair users. By estimation, at most one house out of ten in both settlements is within walking distance of about 500 m to any of the nearest bus stops. No buses or other public transport vehicles have priority seating areas for older people.

The indicator values also revealed major deficiencies in the level of economic security among older slum residents and in their opportunities for engagement in social or communal activities, beyond religious services. There were considerable difficulties with estimating the numbers of reported cases of maltreatment of older persons in Viwandani given a lack of age-disaggregation in official records at the chief’s office and in police occurrence books. In Korogocho, approximately 15 reports of such maltreatment (land conflicts, rape cases, financial disputes involving children/relatives, general harassments by relatives and neighbours, etc.) were estimated to reach the chief’s office on a daily basis. An absence or lack of ready access to electoral records hampered the measurement of older slum residents’ participation in decision-making. However, in both communities the area chiefs estimated that a large majority (80% or more) of older adults had voted in the last general election. Such high participation appears promising. However, perspectives raised in the focus group discussions suggest that it may be more reflective of coercion by influential politicians than of older adults’ own volition—and symptomatic of broader shortcomings in older slum residents’ access to constructive engagement with local government toward addressing needs for change.

Information on community-based health and social care services for older adults with personal care or assistance needs was hard to locate. While a small number of local initiatives exist to support older people—in particular the Korogocho Elders Development Group, and the Catholic Cheshire Home, which houses destitute older people—there is no robust data on the level of care needed in the older slum population and the degree to which it is being met. However, given the earlier mentioned undersupply and lack of access even of basic health services, one may speculate that access to long-term care services is equally, if not more, deficient.

Two ostensibly favourable indicators were housing affordability and quality of life. Half of older-person households were found to spend less than 30% of their income on rent, and a third of older residents rated their overall QoL as good or very good, while a majority reported satisfaction with their living conditions and personal relationships. Especially the latter appears incongruent with the earlier mentioned intergenerational strain that has emerged prominently in qualitative explorations. Upon closer examination, however, the quantitative measures emerge as less positive. Further analysis of NUHDSS data shows that those who spent less of their income on rent tended to be home-owners whose dwellings are of inferior quality to rental properties. Similarly, perspectives raised in the focus group discussions suggest that the apparent high levels of satisfaction may reflect a possible social desirability bias or perceptions of relative improvements to slum environments, for example, as a result of slum upgrading measures [7], rather than actually favourable circumstances.

The equity analyses found a number of indicators—such as on volunteering or participation in local decision-making—to show no marked disparities by gender, age or socio-economic group. Several other indicators, however, do—with gender emerging as the most pronounced axis of inequality and with most identified differentials in line with expected directions of (dis)advantage. Thus, older men are found to be distinctly better off than older women in terms of employment and overall quality of life. However, an exceptional advantage of older women is participation in social groups, as other studies have found [37]. Younger old adults are advantaged vis-a-vis the older old with regard to employment, social group engagement, and quality of life. Finally, the non-poor experience a more favourable employment situation and social group participation compared to the poor.

## 4. Discussion

Taken together, the above review of evidence on likely central challenges to older slum dwellers’ health in Viwandani and Korogocho, and on the poor level of “age friendliness” of the two communities as measured against proposed AFC core indicators suggest a number of perspectives in response to the two key queries we pose in this paper.

### 4.1. What Particular Health-Related Challenges May a Slum-Focused Age-Friendly Cities Initiative in SSA, Need to Consider?

Our evidence points to a number of unsurprising, as well as less obvious, major adversities that such an initiative may possibly need to consider.

The former include, first, entirely adverse physical and built environments in terms of both access and mobility for older adults with capacity limitations, a deficient quality of shelters, and limited access to sound, basic amenities. In many senses such deficiencies may be seen as what define slum environments, at least in SSA. Second, and similarly unsurprising, is an extensive economic insecurity among older adults, which exists despite their broad access to labour opportunities—and thus reflects the pervasiveness of poorly paid, insecure, informal sector work in these communities [38]. Third is a profound lack of access to required, even basic, health services that are able to offer effective responses on the management of, especially, cardiovascular conditions and musculoskeletal disease and their impacts on function. At a structural level such an access gap reflects not only the ‘informality’ of slum settlements per se but, also, as broader SSA perspectives suggest, a continued orientation of the region’s health systems toward serving children or reproductive age adults with little capacity or strategic focus to address old age-related health needs. Importantly, such an orientation extends to emerging SSA agendas on non-communicable diseases. Such agendas centre on the primary, lifestyle-based prevention of the “big four” conditions, with little focus on the management of established conditions or on disorders such as MSK and mental ill-health [9,39].

Equally important as the above are a set of less obvious health adversities. Chief among them are the mental and emotional stress experienced by older slum dwellers, engendered by strained relations with their children or grandchildren or a deep anxiety over these young people’s constrained opportunities and prospects for decent education, work, and fruitful lives. That such adversity is not captured in the quantitative results points to its likely sensitive nature and possible reluctance on the part of older slum residents to declare it—given social norms in SSA settings about the meaning of lacking support and love from younger generation kin [40].

This challenge points to a need for careful efforts to illuminate critical linkages that can exist between the lives and well-being of older and younger generations within slum settings. Indeed, the importance of considering the “linked lives” of old and young is emphasised as a key concept in life course perspectives on ageing [41,42]. The scope of such links may be particularly profound within slum settings given the sheer lack of physical space between generations, both within and outside of dwellings.

A second key, possibly counterintuitive, challenge given the overall deprivation of slum communities, are the pronounced disparities in opportunities and well-being that exist within the older slum population. Such inequalities emerge along gender, socio-economic, and age axes, with women, relatively poorer, and relatively older individuals typically at a disadvantage. The disparities align with observed gender inequalities in health status and functional status within the older slum population [43,44] and resonate with a broader recognition of slums as among the most unequal settlements in the world [45].

A final element that likely underpins many, if not most of the above challenges, are the apparently constrained opportunities of older residents to participate constructively in local decision-making and agenda setting. Such constraints reflect broader shortcomings in local governance participation and mechanisms also in other SSA settings [45].

### 4.2. May the AFC Approach Offer an Appropriate Basis, and the Suggested Core Indicators and Domains a Useful Frame for Initiatives to Advance the Health and Well-Being of Older Adults Living in SSA Slums?

The AFC approach, with its broad focus on equity, the built environment, social relations, engagement across ages, responsive services, and economic wellbeing, certainly captures the principal domains within which older slum residents’ core health-related concerns arise. Its emphasis, moreover, on involving older residents in the identification of local problems and solutions speaks to a likely desire among older slum dwellers to engage with local governance to affect change. There is little question, therefore, that the AFC approach offers a valuable basis for initiatives to promote the health and well-being of older adults in SSA slums.

In their present formulation, however, the specific domains reflected in the AFC indicators capture only partially the likely central challenges that constrain older slum dwellers’ well-being in slum settings. Omitted, for example, are challenges to older adults’ mental and emotional well-being that arise from the intergenerational linkages between the lives of old and young, the profoundly circumscribed quality of housing and basic amenities, and the limited access to even basic health care for chronic conditions.

However, AFC domains and indicators can be adapted to meaningfully and effectively capture the core health-related concerns of older slum residents in SSA. In fact, the identified gaps in the framework as currently articulated underscore a broader point, also highlighted in the indicator pilot study: namely, that there can be no gold standard AFC approach or metrics across settings [26]. Rather, innovation, which builds on local knowledge and other assets—including robust evidence on family and community dynamics and experiences of later life, is required in each locality to tailor a fully-responsive framework of AFC domains and indicators. For SSA slums, an extensively modified framework may be needed.

Both the importance and the opportunity for a community-based ‘age friendly’ initiative are likely particularly pronounced in slums. While the pervasive, multi-faceted deprivation faced by older slum dwellers suggests a heightened need for such an effort, the intimately-shared physical and social space in such settlements may facilitate neighbourhood action and offer increasing returns to investments to promote healthy ageing [4]. A particular opportunity might exist, moreover, for pursuing an integration of an AFC lens within ongoing or future slum upgrading measures, as are envisaged as part of the New Urban Agenda. For example, a standard upgrading of roads and paths may be extended to enhance their walkability [16]—thus lessening constraints on older residents’ mobility. Explorations of how required AFC responses may be considered in the further development of old age focused social protection schemes in SSA countries [29] may be similarly fruitful.

## 5. Conclusions

Age-friendly cities (AFC) initiatives are underway in many parts of the world, mainly in high-income countries. In low and middle income countries, especially in SSA, which are characterized by youthful populations and rapid, largely unplanned, urban expansion, particularly in informal settlements [3], the age-friendly approach is yet to be embraced.

The perspectives offered in this paper have drawn on evidence on key health challenges of older residents, and an application of AFC indicators and domains in two slum communities in Nairobi, Kenya. Our findings suggest a particular need and opportunity for considering and pursuing a slum-focused, age-friendly cities initiative as part of a further expansion of the AFC movement globally.

A pursuit of such an “age-friendly slums” effort will require a possibly substantial modification of the extant AFC framework of dimensions and indicators to fit locally defined, priority challenges and contexts of older adults in slum settings. These priorities must be identified based on focused, participatory explorations involving older slum residents and other stakeholders. Our findings on specific gaps and suggested areas for adaptation in the AFC framework may offer points of reference or working hypotheses for further research and explorations in different slum communities.

At a policy level, a pursuit of an age-friendly slums initiative may offer a lens and vehicle for advancing explicit debate and action on older adults in informal settlements as part of the unfolding slum health [4] and healthy ageing agendas [8,15]. Such action would serve, simultaneously, to advance paramount Africa-regional and global development and urban agendas to which SSA countries have committed. Advocacy efforts to underscore governments’ obligations in these regards ought, therefore, to accompany any steps toward fostering age-friendly slums in the region.

## Figures and Tables

**Table 1:** Age-friendly city core indicator assessment in Korogocho and Viwandani, Nairobi, Kenya, 2015.

Indicator	Suggested Definition ^1^	Operational Definition	Indicator Value	Data Source
Neighbourhood walkability	Proportion of streets in the neighbourhood that have pedestrian paths which meet locally accepted standards.	Same definition	0% (=0/40)	On-the-ground observation, mapping; FGD
Accessibility of public spaces and buildings	Proportion of new and existing public spaces and buildings that are fully accessible by wheelchair.	Same definition	0% (=0/20)	On-the-ground observation, mapping; FGD
Accessibility of public transportation vehicles	Proportion of public transport vehicles with designated places for older people or people who have disabilities.	Same definition	0%	On-the-ground observation, mapping; FGD
Accessibility of public transportation stops	Proportion of housing within walking distance (500 m) to a public transportation stop.	Same definition	10% (Viwandani) <10% (Korogocho)	On-the-ground observation, mapping; FGD
Affordability of housing	Proportion of older people who live in a household that spends less than 30% of their equalized disposable income on housing.	Proportion of households that report spending less than 30% of their income on rent.	51.3% (=683/1332)	NUHDSS
Positive social attitude toward older people	Number of reported cases of maltreatment of older persons (as a proportion of the total number of older people).	Same definition	Inconclusive	On-the-ground observation, mapping
	Proportion of older people who feel respected and socially included in their community.	Proportion of older people who report being “Very satisfied” or “Satisfied” with their personal relationships.	83.6% (=472/565)	UPHD Survey
Engagement in volunteer activity	Proportion of older people who report engaging in volunteer activity in the last month on at least one occasion.	Proportion of older people who report that they have worked with other people in their neighbourhood to fix or improve something or resolve a community issue “Almost daily”, “Once or twice a week”, or “Once or twice per month” in the last four months.	15.6% (=88/565)	UPHD Survey
Paid employment	Proportion of older people who report to have opportunities for paid employment.	Proportion of older people who report being involved in an income generating activity.	74.6% (=994/1332)	NUHDSS
Engagement in socio cultural activity	Proportion of older people who report participating in socio-cultural activities at their own discretion at least once in the last week.	Proportion of older people who report that they have attended any group, club, society, union or organizational meeting “Almost daily”, “Once or twice a week”, or “Once or twice per month” in the last four months.	27.6% (=156/565)	UPHD survey
		Proportion of older people who report attending religious services, other than weddings and funerals, “More than once per week” or “Once per week”.	75.4% (=425/565)	UPHD survey
Participation in local decision making	Proportion of eligible older voters who voted in the most recent local election or legislative initiative.	Same definition	Inconclusive	On-the-ground observation, mapping, consultation
	Proportion of older people who report being involved in decision making about important political, economic, and social issues.	Proportion of older people who report that they have worked with other people in their neighbourhood to fix or improve something or resolve a community issue ‘Almost daily’, ‘Once or twice a week’, or ‘Once or twice per month’ in the last four months.	15.6% (=88/565)	UPHD survey
Availability of information	Proportion of older people who report that local sources of information about their health concerns and service needs are available.	Proportion of older people who did not get healthcare when they needed it in the past 12 months.	42.8% (=242/565)	UPHD survey
Availability of social and health services	Number of older persons with personal care or assistance needs receiving formal (public or private) home-based services.	Same definition	Inconclusive	On-the-ground observation, mapping
Economic security	Proportion of older people who report having had enough income to meet their basic needs over the previous 12 months without public or private assistance.	Proportion of older people who report that they ‘Completely’ or ‘Mostly’ have enough money to meet their basic needs.	11.9% (=67/565)	UPHD Survey
Quality of Life	Proportion of older people who rate their overall QoL as very good (5) or good (4) on a scale ranging from Very poor (1) to Very good (5).	Proportion of older people who rate their overall quality of life “Very good (1)” or “Good (2)”.	33.3% (=188/565)	UPHD Survey
		Proportion of older people who report being “Very satisfied” or “Satisfied” with their personal relationships.	83.6% (=472/565)	UPHD Survey
		Proportion of older people who report being “Very satisfied” or “Satisfied” with the conditions of their living place.	55.3% (=312/565)	UPHD Survey

^1^ Two operational definitions were suggested in the guide for each indicator. In some cases, only one could be applied. This table shows those which were actually used or approximated in this study. FGD: Focus Group Discussions; NUHDSS: Nairobi Urban Health and Demographic Surveillance System; UPHD: Urbanization Poverty and Health Dynamics Study.
